# Ultra-Widefield Retinal Optical Coherence Tomography (OCT) and Angio-OCT Using an Add-On Lens

**DOI:** 10.3390/diagnostics15131697

**Published:** 2025-07-03

**Authors:** Bartosz L. Sikorski

**Affiliations:** 1 Department of Ophthalmology, Nicolaus Copernicus University, 9 M. Sklodowskiej-Curie St., 85-094 Bydgoszcz, Poland; sikorski@doctors.org.uk; 2 International Centre for Translational Eye Research (ICTER), Institute of Physical Chemistry, Polish Academy of Sciences, Kasprzaka 44/52, 01-224 Warsaw, Poland

**Keywords:** OCT, OCT angiography, angio-OCT, widefield retinal imaging, UWF, diabetic retinopathy, branch retinal vein occlusion, central retinal vein occlusion, BRVO, CRVO, optical coherence tomography angiography, REVO FC 130, add-on lens

## Abstract

**Purpose:** This study aims to evaluate the clinical utility of a prototype ultra-widefield (UWF) single-capture optical coherence tomography (OCT) lens developed to image large areas of the retina. **Material and Methods:** This study included OCT and angio-OCT measurements performed with a REVO FC 130 (Optopol Technology, Poland) with an add-on widefield lens in a case series of 215 patients with retinal pathologies and 39 healthy subjects. The imaging width provided by the lens was 22 mm (covering a 110-degree field of view), while the scanning window height ranged from 2.8 to 6 mm. **Results:** The quality of the peripheral UWF OCT and angio-OCT images obtained by REVO FC 130 with the attachable lens is very good and sufficient for patient diagnosis, follow-up, and treatment planning. Both the boundaries of the non-perfusion zones and the location and extent of vascular proliferations can be accurately traced. Furthermore, the vitreoretinal interface can also be accurately assessed over a large area. The imaging quality of the macula with UWF OCT angiography is also good. The mean thickness measurement difference between a UWF and a standard 10 mm 3D retinal scan in a healthy individuals for the Central ETDRS sector was −1.37 ± 2.96 µm (the 95% limits of agreement (LoA) on Bland–Altman plots ranged from −6.82 to 2.43); for the Inferior Inner sector, it was −2.81 ± 1.09 µm (95% LoA, −4.94 to −0.68); for the Inferior Outer sector, it was −1.31 ± 2.58 µm (95% LoA, −6.38 to 3.75); for the Nasal Inner sector: −1.46 ± 1.19 µm (95% LoA, −3.79 to 0.88); for the Nasal Outer sector, it was −0.56 ± 2.61 µm (95% LoA, −5.67 to 4.55); for the Superior Inner sector, it was −2.71 ± 3.16 µm (95% LoA, −8.91 to 3.48); for the Superior Outer sector, it was −1.82 ± 1.39 µm (95% LoA, −4.55 to 0.91); for the Temporal Inner sector, it was −1.77 ± 2.24 µm (95% LoA, −6.16 to 2.62); for the Temporal Outer sector, it was −3.61 ± 1.43 µm (95% LoA, −6.41 to −0.81). **Discussion:** The proposed method of obtaining UWF OCT and UWF angio-OCT images using an add-on lens with the REVO FC 130 gives high-quality scans over the entire 110-degree field of view. This study also shows a high agreement of the ETDRS sector’s thickness measurements between UWF and standard retinal scans, which allows UWF to be used for quantitative macular thickness analysis. Considering its image quality, simplicity, and reliability, an add-on lens can be successfully used for the UWF OCT and OCT angiography evaluation of the retina on a daily basis.

## 1. Introduction

The advent and subsequent development of optical coherence tomography (OCT) has given ophthalmologists the ability to visualise and assess the microstructure of the retina [1,2]. Due to its non-invasiveness and simplicity of performance, OCT has gradually become one of the most commonly performed examinations in ophthalmology. Each successive generation of OCT devices (representing time-domain, spectral-domain, and swept-source OCT) brought both higher imaging resolution and faster scanning [3,4]. Thus, the accurate OCT assessment of the structure of the central part of the retina, i.e., the macula, has now become the gold standard in everyday clinical practice.

OCT examination itself also evolved, and OCT angiography was then developed on its basis [5,6]. The latter made it possible to visualise the retinal and choroidal vascular network non-invasively for the first time. This provided insight into blood flow in the posterior pole of the eye, which can be obtained routinely in almost all patients [7,8].

Once the information about the structure and blood flow in the central part of the retina was thoroughly understood and used in clinical practice, there was a natural interest in scanning a larger area of the fundus. Hence, mosaics consisting of several separate OCT measurements began to be created [9,10]. However, due to the multiple scanning and mechanical repositioning of the scanning head between measurements, examination is very prolonged and thus cannot be performed routinely on every patient [11]. In addition, merging multiple images into one mosaic often caused distortions and discontinuities. The solution to this problem was the advent of the Swept-Source OCT device capable of ultra-widefield (UWF) scanning (Xephilio OCT-S1, Canon, Tokyo, Japan) [12,13]. In this way, data could be collected from a large area of the retina in a single measurement. A further development of this idea is the attempt to use an existing device with an attachable widefield lens (REVO FC 130, Optopol Technology, Poland). Given that the possibility of the widespread use of an imaging technique is key to its adoption, the last approach seems particularly interesting. This is because it does not require the replacement of the OCT device but only needs to extend the device with an add-on widefield lens. As this is a more cost-effective alternative, it may encourage other OCT manufacturers to introduce a similar solution and many ophthalmologists to start using UWF OCT and angio-OCT imaging in daily practice.

The aim of this study is to evaluate the clinical usefulness of a prototype single-capture UWF OCT lens developed for the REVO FC 130 to image large areas of the retina.

## 2. Materials and Methods

This study included OCT and angio-OCT measurements performed in 215 patients (225 eyes, of which 129 were right eyes; mean age 59.3 ± 15.8 years; 126 females) with retinal pathologies. Eight subjects with significant opacification of the ocular media that markedly reduced the quality of retinal imaging were excluded from this study. In addition, 39 healthy subjects (39 eyes, of which 20 were right eyes; mean age 31.1 ± 5.3 years; 20 females) were examined to compare retinal thickness measurements between a UWF and a standard retina scan (Retina 3D).

**Figure 1:**
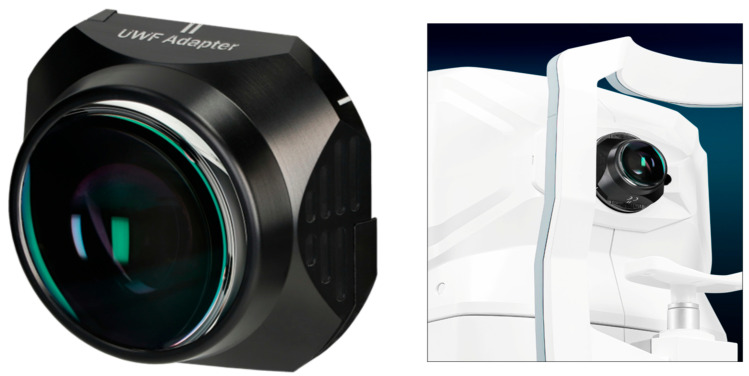
The add-on UWF adapter (**left**) and the adapter mounted on the device (**right**).

The REVO FC 130 spectral-domain OCT device (12.5 beta software version) was used, together with a prototype (which is the final commercial product) single-capture widefield add-on adapter (Figure 1), which consists of two lenses that change the course of the light rays from their original path. The maximum imaging width provided by the adapter is 22 mm. This corresponds—depending on the method of calculation—to a field of view of 73 degrees (expressed as a conventional visual angle defined by the ISO standard and manufacturers of traditional fundus cameras) or 110 degrees (expressed as an eye angle defined by manufacturers of some widefield systems, such as the Optos (Optos, Marlborough, MA, USA), Retcam (Clarity Medical Systems, Pleasanton, CA, USA), and Clarus 500 (Carl Zeiss Meditec, Inc., Dublin, CA, USA) [14]. The field of view is equal in the horizontal and vertical directions. The scanning window height is ~2.8 mm in the default mode and ~6 mm in the full-range mode. The UWF lens is small, attaches quickly to the device with a bayonet mount, and is intuitive to use. It is mounted with just three fingers. This process begins by aligning the lens marker with the device marker. Then, the lens is rotated clockwise by 45 degrees to its final position.

All patients with retinal pathologies had a 3D OCT scan (the number of A-scans in a single B-scan ranged from 512 to a maximum of 2048), with additional radial (eight measurements each of 1024 to 12,288 A-scans) and single linear (consisting of 10 to 100 repetitions, each of 1024 to 12,288 A-scans) scans performed as required. For comparison, patients were also scanned without an add-on lens with a typical scan width (i.e., 6 to 15 mm). All OCT tomograms met the manufacturer’s recommended quality index (QI) cut-off range, which was equal to or greater than 4 for both standard and UWF scans. As the QI used in the REVO FC 130 is a numerical value based on a combination of image intensity and signal-to-noise ratio, for scans with an additional lens, the QI was adjusted for the characteristics of UWF tomograms, in which the area with signal has a different proportion than on a standard retinal scan. The UWF angio-OCT examination was performed at a resolution of 768 × 768 pixels. To partially compensate for the lower density of wider scans, the number of A and B-scans in the UWF examinations was increased compared to standard scanning protocols.

Retinal thickness measurements between UWF and a standard retina scan (Retina 3D) were compared in nine ETDRS sectors (Central, Inferior Inner, Inferior Outer, Nasal Inner, Nasal Outer, Superior Inner, Superior Outer, Temporal Inner, and Temporal Outer). The UWF scan covered an area of 22 × 22 mm (256 B-scans × 1280 A-scans), while the Retina 3D scan imaged an area of 10 × 10 mm (168 B-scans × 1024 A-scans). To assess agreement between the UWF and standard retinal scan thickness profile, the paired sample *t*-tests and Bland–Altman analyses were performed, and 95% limits of agreement were calculated by the mean difference ±1.96 SD. A *p* value of less than 0.05 was considered statistically significant. Statistical analysis was performed using Statistica 13.1 (Dell Inc., Round Rock, TX, USA).

This study was conducted with the approval of the Bioethics Committee in accordance with the Declaration of Helsinki. All participants signed an informed consent.

This study attempted to answer the following questions: What is the quality of widefield scanning with the add-on lens? What is the quality of the centre of the widefield angio-OCT scan compared to regular scans obtained without the lens? What is the practical utility of such scanning?

## 3. Results

A selection of UWF OCT and angio-OCT case series measurements is presented below. The characteristic features of the images are pointed out, using them as examples.

### 3.1. OCT Measurements

UWF OCT imaging provides us with information about the morphology of the retina and adjacent structures outside the central part of the posterior pole.

**Figure 2:**
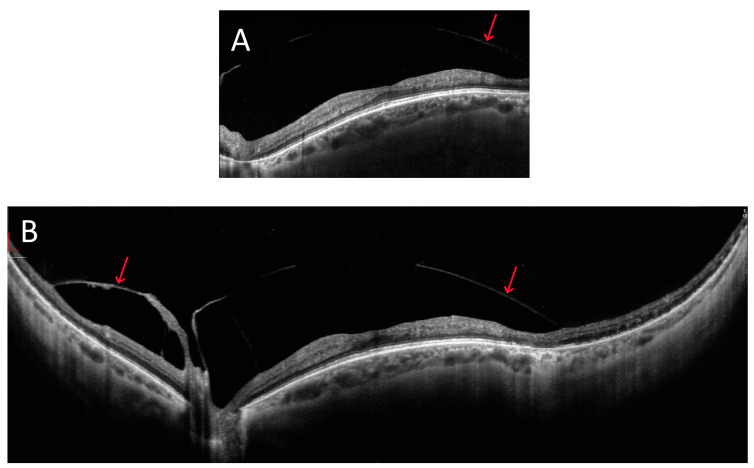
OCT image of a patient with a dome-shaped macula (female, age 46): (**A**) 10 mm wide scan; (**B**) 22 mm wide scan. The arrows point at the vitreoretinal traction.

Figure 2 presents a dome-shaped macula. A 10 mm wide horizontal cross-section through the macula shows its slightly convex shape. An ultra-wide 22 mm scan clearly emphasises this shape. In addition, it shows the vitreoretinal traction (arrows), which is fully captured in a single image. Temporal to the macula, it can be seen that this traction pulls the retina, leading to schisis.

**Figure 3:**
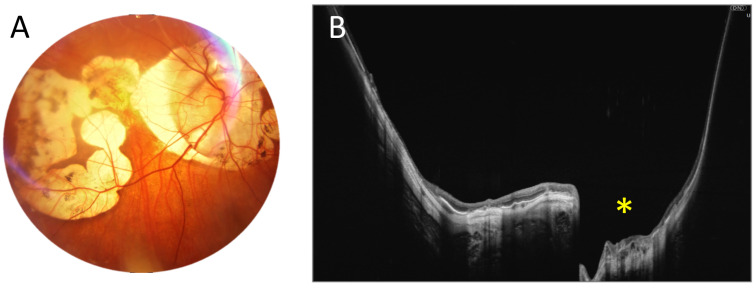
Posterior staphyloma due to high myopia (male, age 43). (**A**). Colour fundus photograph showing extensive atrophy. (**B**). A 22 mm wide UWF full-range OCT scan of the posterior pole. The asterisk indicates a localized outpouching around the optic disc.

Another figure (Figure 3) demonstrates posterior staphyloma due to high myopia, which extends both vertically and horizontally over a significant area. With a full-range UWF scan that is 22 mm wide and 6 mm high, we can trace the significant depression in the central part of the retina (asterisk) and the strongly curved shape of the posterior pole.

**Figure 4:**
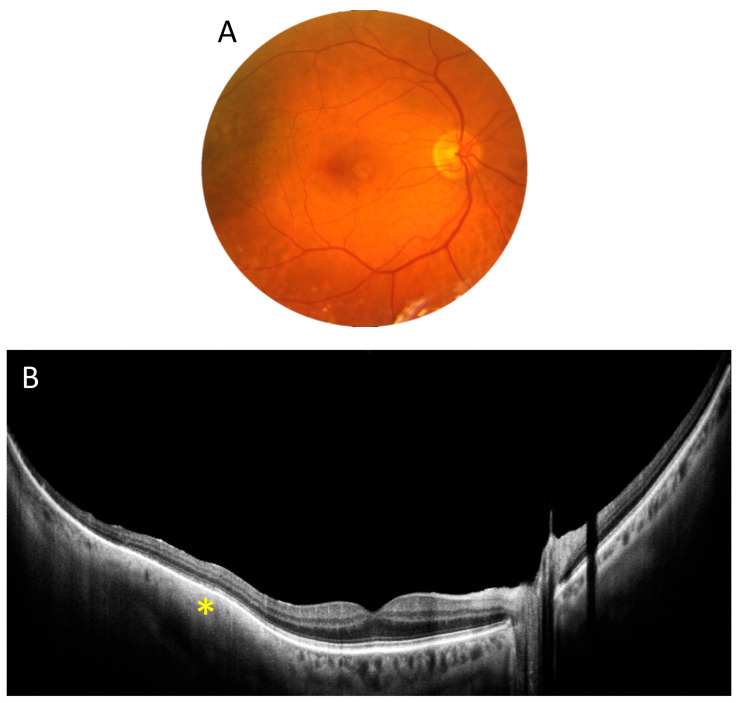
Choroidal tumour (female, age 55). (**A**). Colour fundus photograph revealing a darker area temporal to the macula. (**B**). A 22 mm wide UWF OCT scan of the posterior pole. The asterisk shows a solid choroidal structure.

Figure 4 depicts a patient with a choroidal tumour. The UWF scan reveals a solid choroidal structure (asterisk) with no large vessels, along with overlying drusen temporal to the macula, while the centre of the scan shows a normal retina and choroid.

**Figure 5:**
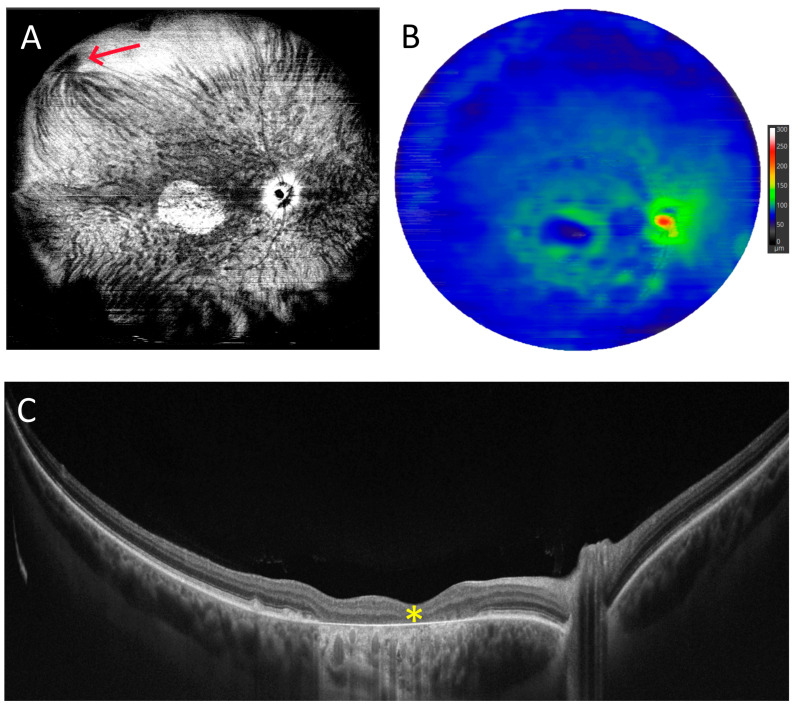
Geographic atrophy in the macula (female, age 66). (**A**). Fundus reconstruction derived from OCT data at the choroidal level. The red arrow indicates the vortex vein. (**B**). UWF OCT outer retina thickness map. (**C**). A 22 mm wide UWF OCT scan. The asterisk shows the central area of atrophy.

**Figure 6:**
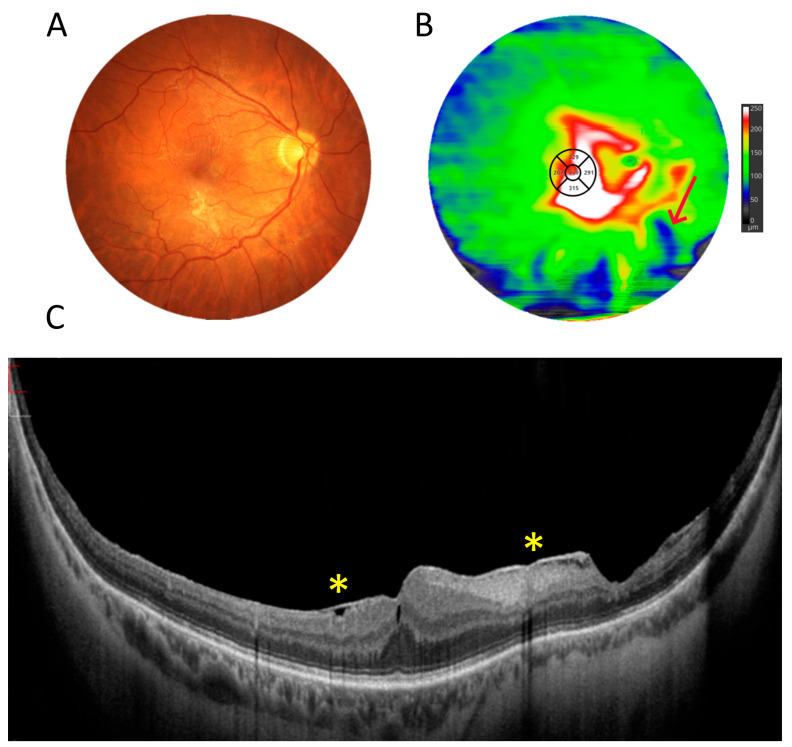
Patient with epiretinal membrane (male, age 62). (**A**) Colour fundus photograph. (**B**) UWF OCT thickness map of the inner retina. The red arrow indicates the site of iatrogenic damage to the retina. (**C**) A 22 mm wide UWF OCT scan. The asterisks indicate epiretinal membrane.

The next two figures illustrate the use of UWF OCT scanning for en-face analysis. In Figure 5, the central area of atrophy (asterisk) can be seen to be surrounded by only a few small drusen. OCT fundus reconstruction at the choroidal level clearly visualises the boundaries of the area of increased light penetration into the choroid at the site of retinal pigment epithelial atrophy. The outer retina thickness map made from OCT data reveals a reduction in the thickness of the retinal layers in the centre. Figure 6 shows the fundus of a patient with epiretinal membrane (asterisks). In this case, by reconstructing the inner retinal thickness map, the extent of the membrane can be visualised in detail. In addition, the post-operative retinal damage is visible as an area of inner retinal atrophy (arrow).

**Figure 7:**
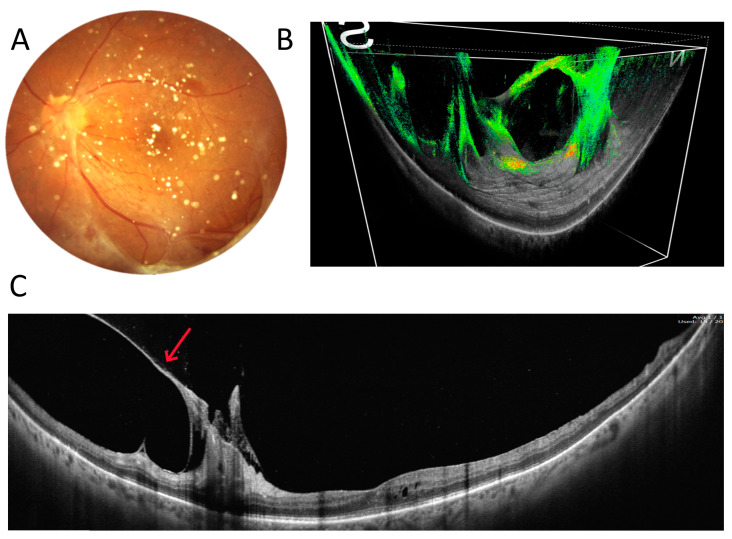
Vitreoretinal traction in proliferative diabetic retinopathy (female, age 53). (**A**) Colour fundus photograph. (**B**) Three-dimensional UWF OCT reconstruction of the interface between the retina and vitreous body. (**C**) A 22 mm wide UWF OCT scan of the retina across the posterior pole. The arrow points at a strongly adherent vitreous traction.

**Figure 8:**
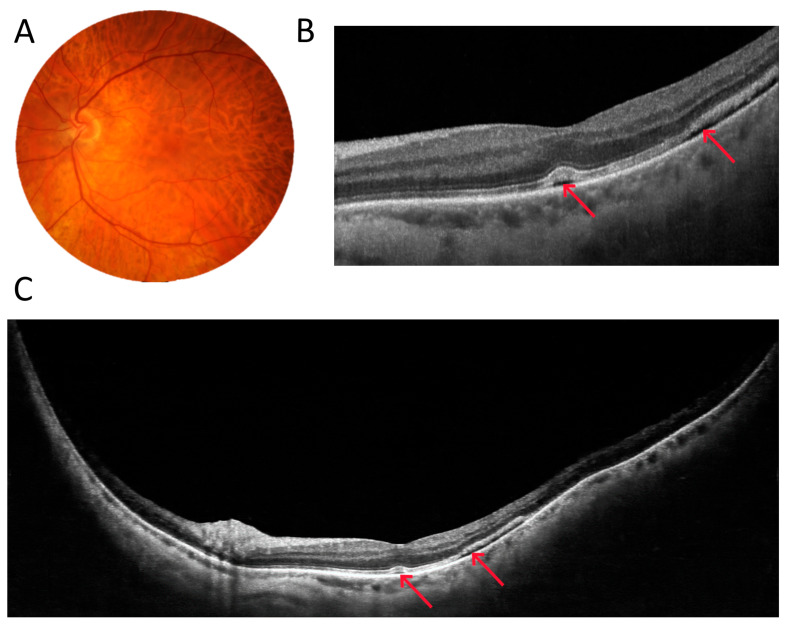
Patient after vitrectomy for retinal detachment (male, age 37). (**A**) Colour fundus photograph. (**B**) A 6 mm wide macular scan showing a flat pocket of fluid in its temporal part. (**C**) A 22 mm wide UWF OCT scan revealing the entire area of sensory retinal detachment. The arrows point at subretinal fluid.

The last two figures in this section demonstrate surgical cases. Figure 7 shows an example of the preoperative assessment of a strongly adherent vitreous traction (arrow) to the optic disc due to proliferative diabetic retinopathy (DR). A three-dimensional reconstruction of the vitreoretinal interface from a 22 mm wide OCT scan visualises the reciprocal relationship of the two structures on a single image. Figure 8, on the other hand, shows the eye after vitrectomy for retinal detachment. A typical 6 mm OCT scan reveals a shallow pocket of fluid (arrows) under the sensory retina in the macula. Unfortunately, such a scan does not show how far into the periphery the fluid extends. It is only with a 22 mm UWF scan that all the fluid can be visualised.

### 3.2. Angio-OCT Measurements

The idea behind UWF angio-OCT imaging is to detect vascular pathologies and changes in blood flow outside the macula. This is particularly important in diseases leading to the development of non-perfusion and vascular proliferation. The most common are DR and retinal vascular occlusion.

**Figure 9:**
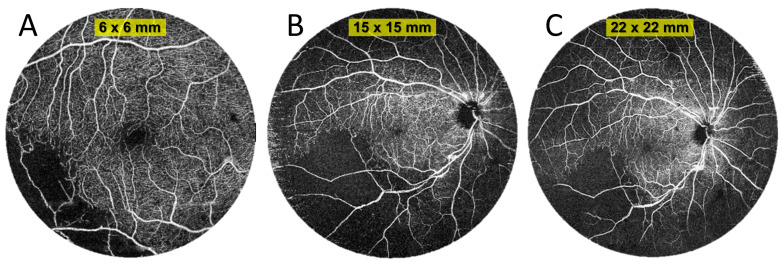
OCT angiography of a patient with branch retinal vein occlusion (female, age 46). (**A**) Measurement with a scan area of 6 × 6 mm. (**B**) Angiography taken over an area of 15 × 15 mm. (**C**) UWF OCT angiography covering an area 22 mm in diameter.

**Figure 10:**
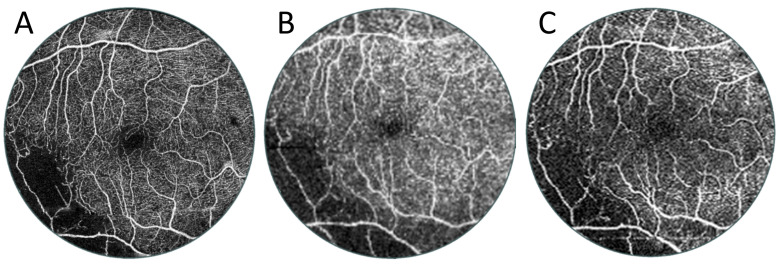
Comparison of the same size central 6 × 6 mm area of different-sized OCT angiographies of the patient in Figure 9. (**A**) Reference OCT angiography of 6 mm diameter. (**B**) The central area of the OCT examination with a width of 15 mm. This angio-OCT examination has a lower scanning density and hence lower quality than the other two images. (**C**) Enlarged UWF OCT centre of angiography taken with a diameter of 22 mm.

Figure 9 shows a case of a patient with branch retinal vein occlusion. A typical 6 × 6 mm scan shows an area of non-perfusion in the lower left of the image. This information enables a diagnosis but not treatment (e.g., in the form of laser therapy). A 15 × 15 mm widefield scan taken without an additional lens, which is the widest possible on the device, shows a slightly larger no-flow area. In contrast, the UWF scan clearly detects extensive ischaemia. This is clinically useful information, as it can be used to guide laser therapy. These angiograms can also be analysed from another perspective. Figure 10 shows a comparison of exactly the same area from scans of different widths of the patient shown in Figure 9. In the case of the 6 × 6 mm scan, this is the entire image, and in the case of the other two scans, it is their central 6 × 6 mm portion. It can be seen that the quality of the routine 6 × 6 mm measurement is slightly higher, but the UWF scan also carries all clinically important information of this area. It allows the boundaries of the zones of non-perfusion in the centre of the posterior pole to be traced and allows an overall vascular assessment of the macula to be performed.

**Figure 11:**
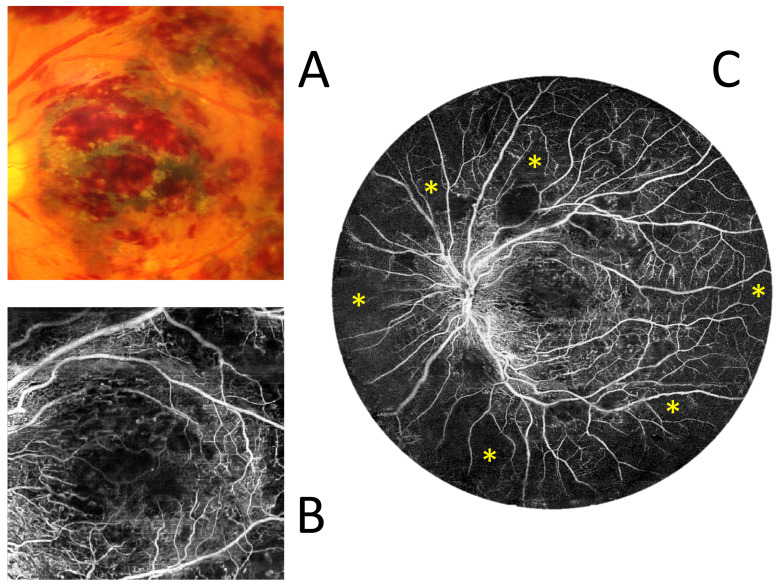
Central retinal vein occlusion (female, age 69). (**A**) Colour fundus photograph of the central 6 × 6 mm area. (**B**) OCT angiography measurement of the size of a colour fundus photograph. (**C**) UWF OCT angiography covering an area that is 22 mm in diameter. The asterisks indicate non-perfusion zones.

Figure 11 shows a case of a patient with central retinal vein occlusion. The prominent haemorrhages obscure the view of the central macula on the colour fundus photo and cast small optical shadows in OCT angiography. The visualisation of the central circulation in a 22 × 22 mm UWF OCT scan is similar to that of a 6 × 6 mm measurement. By using a wide scan, however, extensive zones of non-perfusion (asterisks) throughout the mid-periphery can be easily seen at the same time.

**Figure 12:**
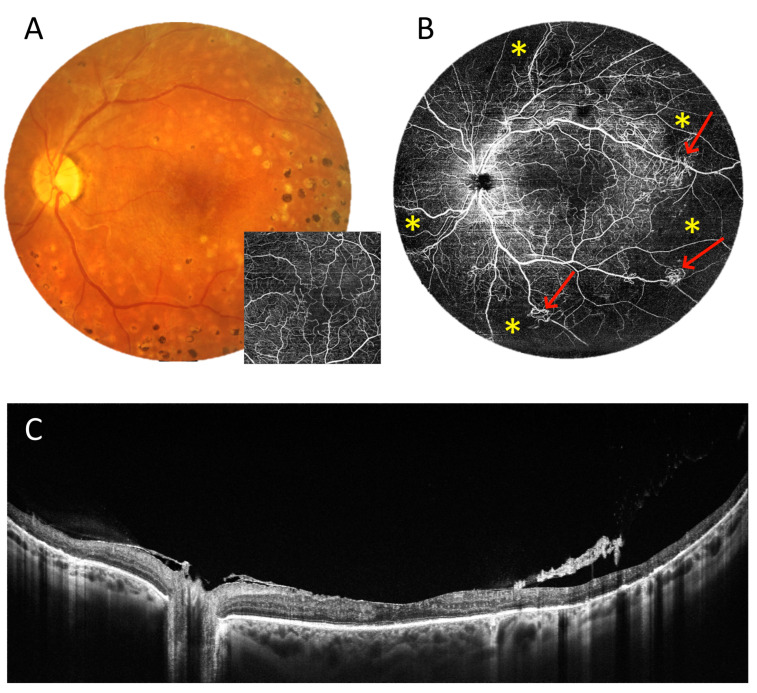
Proliferative diabetic retinopathy (female, age 54). (**A**). Colour fundus photograph and 6 × 6 mm OCT angiography. (**B**). UWF OCT angiography from a 22 mm wide area. The asterisks indicate non-perfusion zones and the arrows point to vascular proliferation. (**C**). UWF OCT scan of the posterior pole.

**Figure 13:**
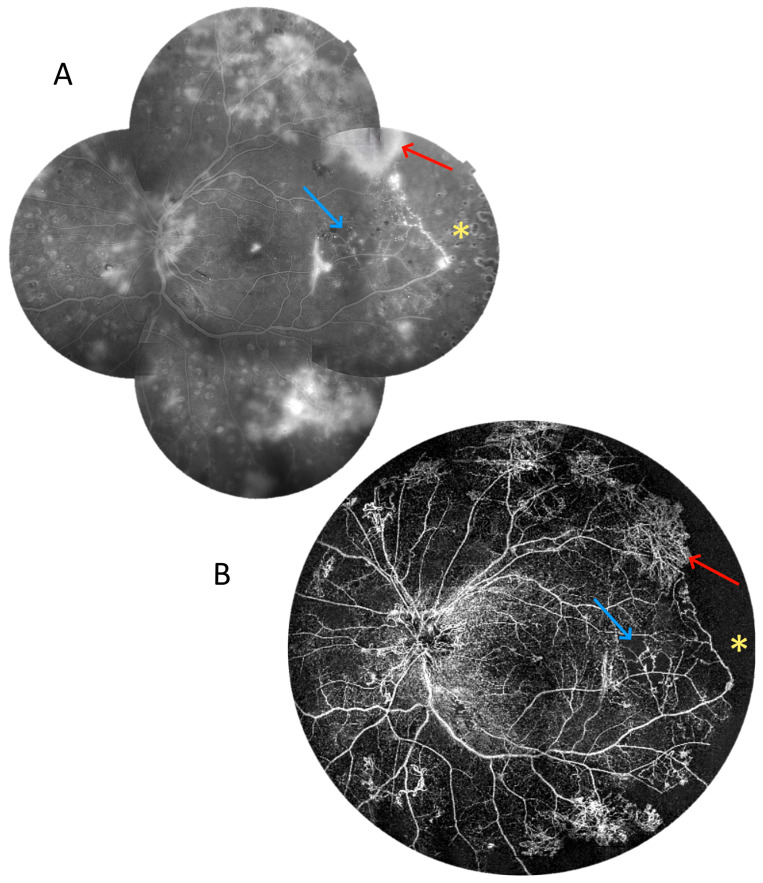
Proliferative diabetic retinopathy (male, age 37). (**A**) A wide-angle fluorescein angiography mosaic. (**B**) UWF OCT angiography covering a 22 mm wide area. The red arrows point at the vascular proliferation, whereas the blue arrows indicate capillary dropout. The asterisks show non-perfusion zones.

The next two cases present patients with proliferative DR. Figure 12 shows a 6 × 6 mm scan of the macula. It presents an enlargement of the foveal avascular zone and small areas of non-perfusion around it. It does not provide any other retinal or flow information. In contrast, the 22 mm UWF scan shows extensive zones of non-perfusion (asterisks) in the periphery and vascular proliferation (arrows), in addition to the above-mentioned changes in the centre. Thus, it indicates an urgent need for extended treatment. Figure 13 relates the 22 mm UWF angio-OCT scan to the wide-angle fluorescein angiography (FA) mosaic. Both examinations reveal vascular proliferation (red arrow), capillary dropout (blue arrow), and non-perfusion zones (asterisk). However, angio-OCT shows vascular structures more clearly due to the lack of background fluorescence, as well as the staining of other lesions.

### 3.3. OCT and Angio-OCT Measurements

**Figure 14:**
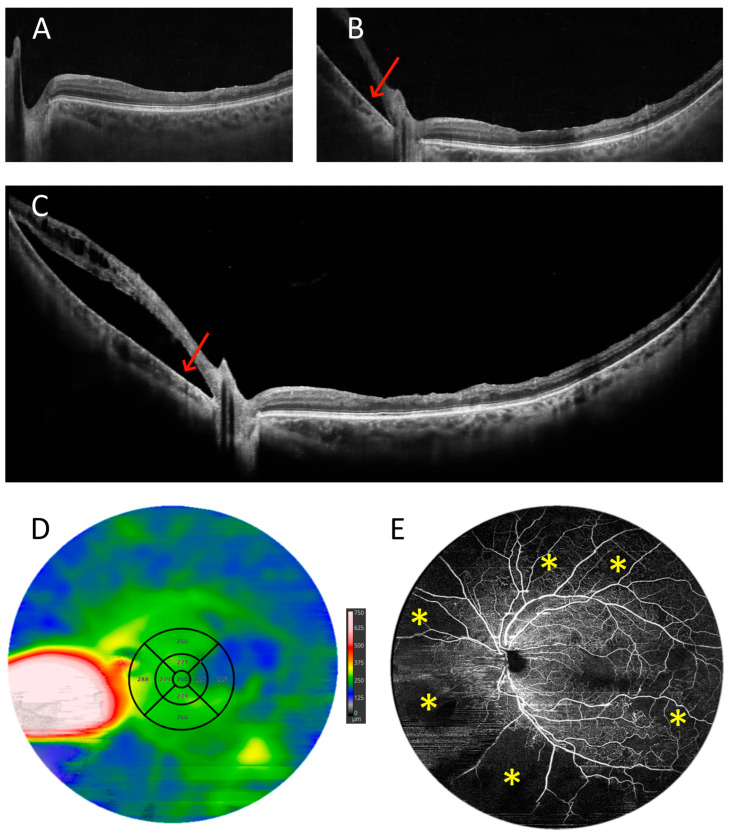
Patient after vitrectomy for retinal detachment (male, age 54). (**A**–**C**) OCT scans with widths of 10, 15, and 22 mm, respectively. (**D**) UWF OCT retinal thickness map. The arrow points at the retinal detachment. (**E**) UWF OCT angiography of the 22 mm area. The asterisks indicate extensive zones of non-perfusion.

Figure 14 shows the imaging findings of a patient who underwent vitrectomy for retinal detachment. A routine 10 mm scan shows nothing but structural abnormalities of the inner layers of the retina and irregularities of its surface. A wider 15 mm scan reveals a retinal detachment (arrow) on the other side of the optic disc, but the extent of the detachment is not visible. Only a UWF measurement allows the whole area of the detachment (arrow) to be visualised. This fact is confirmed by the retinal thickness map, on which the borders of the detachment are clearly visible. In turn, angio-OCT led to the discovery of extensive zones of non-perfusion (asterisks) in this patient.

### 3.4. UWF Angio-OCT Mosaics

**Figure 15:**
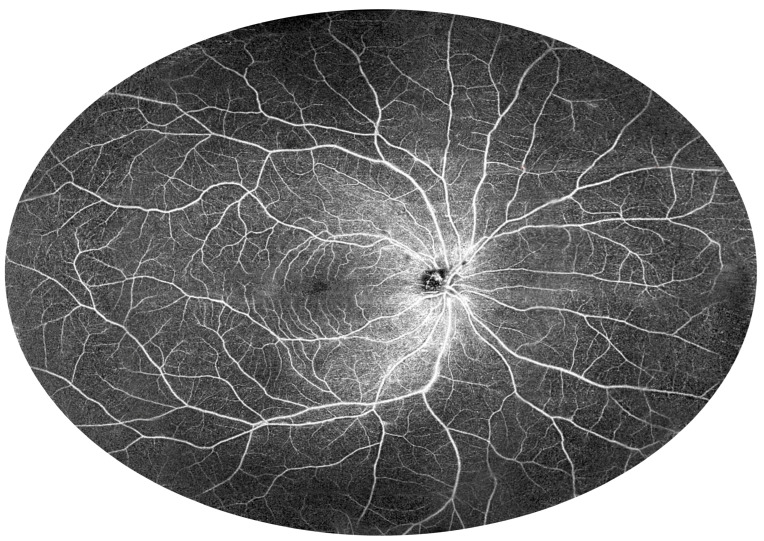
Healthy individual (female, age 26). The figure shows a 140-degree mosaic of 22 mm wide UWF OCT angiography measurements, showing increased imaging field of view (140-degree external field of view is equal to 210-degree internal field of view—nomenclature as in UWF Optos devices).

**Figure 16:**
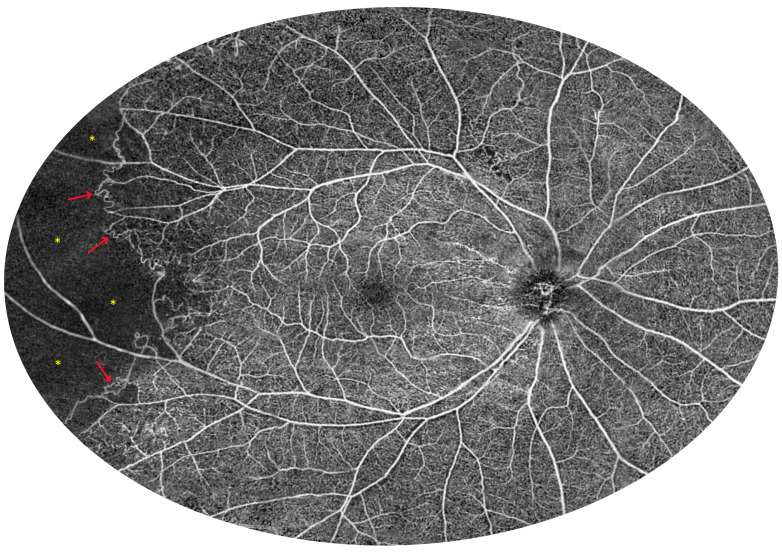
Eales disease (male, age 27). The figure shows a 100-degree OCT angiography montage of 22 mm UWF scans, showing extensive peripheral ischaemia and collateral formation (100-degree external field of view is equal to 150-degree internal field of view—nomenclature as in UWF Optos devices). The asterisks indicate non-perfusion zones and the arrows point at collateral vessels.

UWF imaging can be further expanded by combining UWF measurements from different adjacent locations. Figure 15 shows a mosaic of retinal UWF measurements in a healthy individual. Both macular circulation with foveal avascular zones in the centre and normal vessels at the far periphery are clearly visible. In contrast, Figure 16 shows Eales disease with peripheral ischaemia (asterisks) and collateral formation (arrows) in the temporal part of the image. Several small areas of non-perfusion can also be seen above the optic disc.

### 3.5. Retinal Thickness Measurements

**Figure 17:**
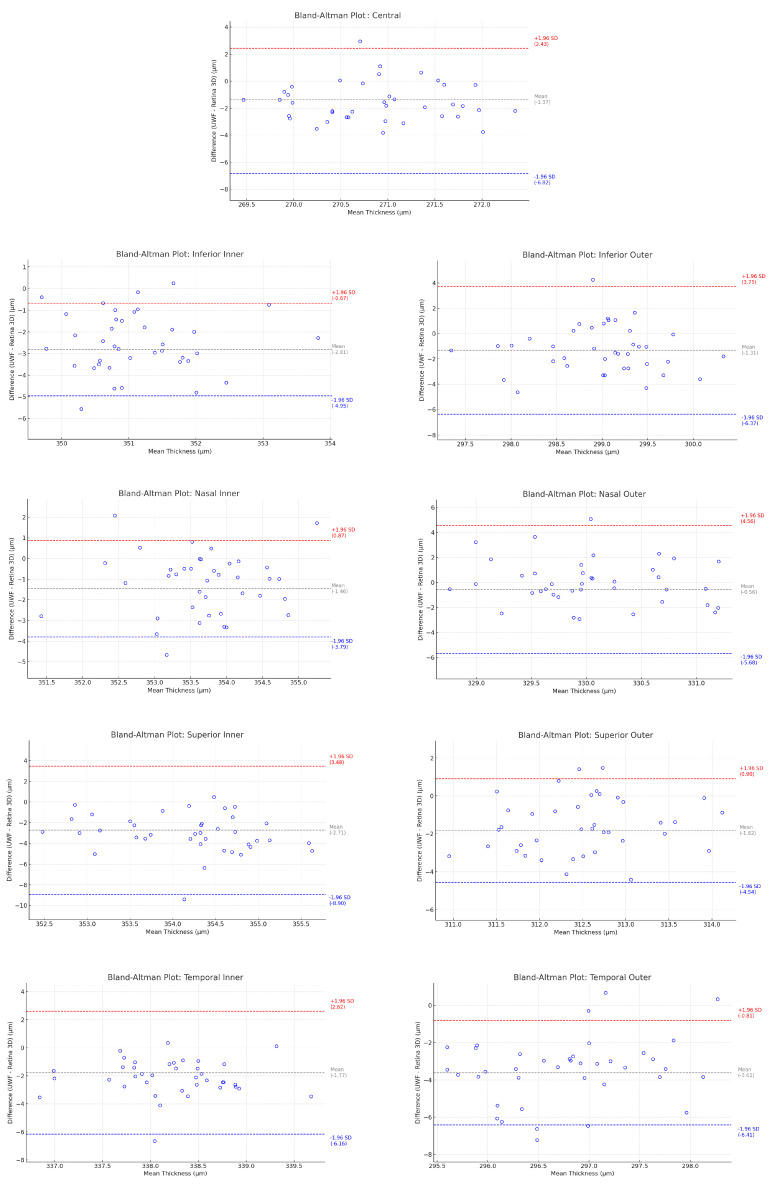
Bland–Altman plots for a thickness profile comparison between UWF and a standard retina scan (Retina 3D) in ETDRS sectors.

The agreement of retinal thickness measurements between UWF and standard 10 mm Retina 3D scans in healthy individuals is shown in Table 1 and Figure 17. The mean thickness measurement difference for the ETDRS sectors is as follows: Central: −1.37 ± 2.96 µm (the 95% limits of agreement (LoA) on Bland–Altman plots ranged from −6.82 to 2.43); Inferior Inner: −2.81 ± 1.09 µm (95% LoA, −4.94 to −0.68); Inferior Outer: −1.31 ± 2.58 µm (95% LoA, −6.38 to 3.75); Nasal Inner: −1.46 ± 1.19 µm (95% LoA, −3.79 to 0.88); Nasal Outer: −0.56 ± 2.61 µm (95% LoA, −5.67 to 4.55); Superior Inner: −2.71 ± 3.16 µm (95% LoA, −8.91 to 3.48); Superior Outer: −1.82 ± 1.39 µm (95% LoA, −4.55 to 0.91); Temporal Inner: −1.77 ± 2.24 µm (95% LoA, −6.16 to 2.62); and Temporal Outer: −3.61 ± 1.43 µm (95% LoA, −6.41 to −0.81).

## 4. Discussion

OCT angiography has been successfully used in the diagnosis, monitoring, and treatment of ocular vascular disease. However, most OCT devices image a relatively small central part of the retinal circulation, beyond which additional flow abnormalities may be present [15]. It has been shown that these peripheral vascular changes may be particularly important as they are associated with both a more advanced form of a disease and a higher risk of disease progression [16,17,18,19,20]. Therefore, it is crucial to be able to image the widest possible area of the retinal circulation.

The use of an add-on lens with the REVO FC 130 provides OCT imaging with a width of 22 mm, which corresponds to a 110-degree field of view. This undoubtedly allows retinal images to be obtained well beyond the vascular arcades. As shown in Figure 5, the vortex veins area (red arrow) can be easily imaged in this way. And by mounting multiple UWF measurements into a single mosaic, the imaging field can be increased even further into the periphery (Figure 15 and Figure 16).

When performing UWF OCT imaging, we want to gain insight into the structure of the retina and its interface with the vitreous body over a large area extending well beyond the macula. At the same time, we do not want to lose the ability to assess the macular area. The same is true for widefield OCT angiography. While visualising peripheral zones of non-perfusion and neovascularisation, we still want to see potential abnormalities in the central part of the retina. Thus, if UWF OCT imaging is to become the standard, it must provide good imaging quality over the entire scanned area. When it comes to the quality of the structural OCT imaging of the macula on widefield scans captured with an add-on lens compared to a typical narrow-field scan covering the macula alone, it is comparable. In the case of OCT angiography, however, the quality is slightly lower due to the lower scanning density. Nevertheless, it is still sufficient for diagnosis and patient care. If there is a need for high-resolution OCT angiography of the macula, an additional narrow-field scan can always be performed. It is worth noting that from a technical point of view, it would be possible to obtain a similar quality of angio-OCT regardless of the scan width by increasing the number of A- and B-scans proportionally. In practice, however, the limitation is the examination time, which cannot become extremely long. Therefore, in the case of UWF angio-OCT, the number of A- and B-scans is only partially increased to find a balance between high scanning densities and long measurement times.

UWF scanning with an add-on lens has proven to be very useful in clinical practice. It allows ophthalmologists to detect pathologies located outside the macula during a single measurement. In this way, unexpected lesions can be detected during a routine examination (e.g., tumours). With UWF angio-OCT, both the diagnosis and treatment of ischaemic and proliferative changes in retinopathies (e.g., DR, post-retinal vascular occlusion, Coats disease, etc.) become much simpler [21]. In particular, the boundaries of non-perfusion zones can be accurately traced. Furthermore, the vitreoretinal–retinal interface can also be accurately assessed over a large area. This is helpful for both the diagnosis and preoperative evaluation of the retina. It can also facilitate assessment after vitrectomy. In this situation, it can, for example, determine the extent of residual lesions such as subretinal fluid or damage to the retinal surface. A particularly interesting example of a surprising finding is shown in Figure 14, as OCT angiography performed during a routine follow-up visit after vitrectomy revealed that the patient had extensive ischaemia, which was not previously suspected. Without UWF OCT angiography, this would not have been discovered.

With UWF OCT angiography, we can hope to develop new tools for assessing the risk of progression and improved criteria for monitoring and treating retinal vascular diseases, particularly DR. The regular-sized OCT angiograms used to date accurately identify vascular proliferations and nonperfusion zones [22], as well as at least some microaneurysms [23]. They are also successfully used to quantify capillary density and the size of non-perfusion areas [24,25,26,27,28]. Taking all this into account, a simple question arises as to why angio-OCT is not then used in clinical practice instead of the photographic classification of DR, which was developed several decades ago. This is partly due to the fact that the imaging performance of earlier OCT angiography devices in DR was not very high, and also, the calculation of vessel density in commercial OCT systems is strongly dependent on signal strength [29,30]. However, with advances in OCT technology and the acquisition of increasingly reproducible scans of higher quality, these issues should no longer be a major obstacle. That being said, the main problem still lies in the fact that the field of view of the angio-OCT used today is too small. As we would like OCT angiography to bring a breakthrough and improve the short-term prediction of vision-threatening DR development compared to colour images, in order to find new biomarkers, its imaging area cannot be smaller than that of fundus images. Furthermore, the current ETDRS classification not only empirically identifies the clinical features associated with the development of proliferative DR but also stratifies the risk into mild, moderate, and severe non-proliferative DR [31]. It is important to realise that just because the measurement of no-flow zones on angio-OCT correlates with non-proliferative features of DR [32,33], it does not necessarily mean that it significantly improves prediction. Indeed, a strong correlation between non-perfusion and DR severity on angio-OCT does not mean that it can automatically be used to make reliable clinical decisions. Only finding the subclinical precursors of progression to proliferative DR on angio-OCT may change the treatment of patients with diabetes. We could, for example, try to identify people with severe non-proliferative DR who will progress to proliferative DR within a year or patients who will develop vision-threatening DR in the short term and treat them. But without reliable, high-quality, and widely used UWF OCT angiography, this will be virtually impossible. Only its widespread introduction into clinical practice can lead to a change in the classification of proliferative DR in the same way that OCT has changed the classification of diabetic macular oedema [4]. Using UWF imaging, we will not only be able to detect vascular proliferation but also to objectively track the response to treatment. In this way, UWF angio-OCT has the potential to revolutionise the way we care for patients with vascular diseases, particularly diabetes, and change the principles of treatment.

It should also be mentioned that UWF angio-OCT has some advantages over conventional FA, which remains a standard in proliferative DR diagnostics. OCT angiography does not use external dye, and hence, there is no choroidal background fluorescence and the interference of hyperfluorescence from dye leakage (Figure 13, red arrow), which can obscure details in FA. As angio-OCT can segment different layers of the retina and choroid, it also enables the visualization of vascular structures while excluding retinal pigment epithelium from analysis. This is particularly important in the case of laser scars, which accumulate dye, thus making it difficult to observe the vascular structures above (Figure 13, asterisk). On the other hand, angio-OCT is not a dynamic examination, nor does it show leakage and some of the smaller structures (e.g., microaneurysms) where blood flow velocity is outside the detection range. Another major drawback is its susceptibility to motion and projection artifacts, which can distort the image, leading to misinterpretation. However, as OCT angiography research progresses, at least some of these problems will gradually be overcome.

A common way to assess the macula over time in OCT is to measure retinal thickness. For the method to be reliable, the OCT tomograms obtained at follow-up visits must be able to measure thickness accurately and in the same way as those taken at the initial examination. In everyday clinical practice, however, the patient is not always examined with the same scanning protocol at each visit. Therefore, it is important that the results of thickness measurements obtained from different types of scans are interchangeable. As the comparison of retinal thickness values in the ETDRS sectors between the UWF and the standard 10 mm scan showed very high agreement, the UWF scans can be used to quantify retinal thickness in the macula in the same way as standard-length scans.

UWF scanning should be a simple and quick examination that does not burden the patient. From the perspective of the healthcare system, it should, in turn, not require a significant financial outlay [34,35]. Only an examination with such features has the potential to be willingly performed on any patient who requires it. The use of a wide-angle lens, which is easily and quickly attached to the OCT instrument, does not differ significantly from scanning with an instrument with such a function built in, as the lens simply becomes part of the optical system of the instrument. An unquestionable advantage of the lens is its cost-effectiveness, especially if one already owns a REVO FC 130 instrument [36].

In comparison with the Xephilio OCT-S1 (Canon, Tokyo, Japan), which has a similar imaging field, the penetration depth of the scanning beam of the REVO FC 130 is slightly smaller, as the latter is a Swept-Source device. On the other hand, the REVO FC 130 scans faster (130,000 vs. 100,000 A-scans per second for the Xephilio OCT-S1). It is also worth noting that other OCT devices on the market provide only slightly wider-than-typical scans, i.e., up to approximately 17 mm wide (PLEX Elite 9000, Carl Zeiss Meditec, Dublin, OH, USA; Spectralis, Heidelberg Engineering, Heidelberg, Germany; Mirante, Nidek Co., Ltd., Gamagori, Japan) [37].

The add-on lens also has minor inconveniences. Firstly, there is the smaller working distance (15 mm), which makes the automatic eye alignment procedure take longer compared to a standard examination. Also, the shorter distance means that, in some rare situations, the lens can be quite close to the eyelid. Secondly, real-time eye tracking is not available for the UWF examination. Instead, iTracking uses a motion correction technique, which requires longer processing times. Also, the live preview of the fundus is smaller than the UWF scan area. Another issue is the limitation of UWF OCT imaging due to the variable curvature of the retina. For any OCT scan, depending on the radius of curvature of the retina, the imaging window can be filled to a different degree. With a large radius, the measured image is limited by the width of the window and by its height, with a small radius. Particularly in the UWF examination, since we are imaging a wider area of the retina, at a small radius of curvature, the retina may extend beyond the upper limit of the imaging window (e.g., in high myopia). In this case, it is helpful to use the full-range mode of the device, which allows the height of the imaging window to be increased from 2.8 to 6 mm (Figure 3).

In conclusion, the proposed method of obtaining UWF OCT and angio-OCT images using an add-on lens with the REVO FC 130 gives high-quality scans over their entire length. Both the information from the centre and the periphery of the scans is clinically useful. Given the good image quality, simplicity, reliability, and cost-effectiveness of such a solution, it can be successfully used for the UWF OCT and angio-OCT evaluation of the retina on a daily basis.

## Figures and Tables

**Table 1:** Agreement of retinal thickness measurements between UWF and Retina 3D scan.

ETDRS Subfields n = 39	Mean UWF Thickness (µm)	Mean Retina 3D Thickness (µm)	Mean Difference (µm)	Mean Difference SD	Lower LoA (µm)	95% CI of Lower LoA	Upper LoA (µm)	95% CI of Upper LoA
Central	270.02	271.39	−1.37	2.96	−6.82	−2.78 to −10.87	2.43	−0.32 to 22.48
Inferior Inner	349.73	352.54	−2.81	1.09	−4.94	−6.42 to −3.45	−0.68	−2.17 to 0.80
Inferior Outer	298.24	299.56	−1.31	2.58	−6.38	−9.91 to −2.85	3.75	0.22 to 7.28
Nasal Inner	352.97	354.43	−1.46	1.19	−3.79	−5.42 to −2.16	0.88	−0.75 to 2.51
Nasal Outer	329.76	330.32	−0.56	2.61	−5.67	−9.24 to −2.11	4.55	0.99 to 8.12
Superior Inner	352.78	355.49	−2.71	3.16	−8.91	−13.23 to −4.59	3.48	−0.84 to 7.81
Superior Outer	311.55	313.37	−1.82	1.39	−4.55	−6.45 to −2.64	0.91	−0.99 to 2.82
Temporal Inner	337.16	338.93	−1.77	2.24	−6.16	−9.22 to −3.10	2.62	−0.44 to 5.68
Temporal Outer	295.19	298.80	−3.61	1.43	−6.41	−8.37 to −4.46	−0.81	−2.76 to 1.15

UWF: Ultra-widefield; LoAs: limits of agreement; CIs: confidence intervals. The UWF and Retina 3D scans covered an area of 22 × 22 mm (256 B scans × 1280 A scans) and 10 × 10 mm (168 B scans × 1024 A scans), respectively.

## Data Availability

Dataset available on request from the author.
